# Evaluation of procalcitonin for diagnosis of neonatal sepsis of vertical transmission

**DOI:** 10.1186/1471-2431-7-9

**Published:** 2007-02-26

**Authors:** José B López Sastre, David Pérez Solís, Vicente Roqués Serradilla, Belén Fernández Colomer, Gil D Coto Cotallo, Grupo de Hospitales Castrillo

**Affiliations:** 1 Service of Neonatology, Hospital Universitario Central de Asturias, Oviedo, Spain; 2 Service of Neonatology, Hospital Universitario La Fe, Valencia, Spain; 3 Grupo de Hospitales Castrillo. Service of Neonatology, Hospital Universitario Central de Asturias, Oviedo, Spain

## Abstract

**Background:**

The results of recent studies suggest the usefulness of PCT for early diagnosis of neonatal sepsis, with varying results. The aim of this prospective multicenter study was to determine the behavior of serum PCT concentrations in both uninfected and infected neonates, and to assess the value of this marker for diagnosis of neonatal sepsis of vertical transmission.

**Methods:**

PCT was measured in 827 blood samples collected prospectively from 317 neonates admitted to 13 acute-care teaching hospitals in Spain over one year. Serum PCT concentrations were determined by a specific immunoluminometric assay. The diagnostic efficacy of PCT at birth and within 12–24 h and 36–48 h of life was evaluated calculating the sensitivity, specificity, and likelihood ratio of positive and negative results.

**Results:**

169 asymptomatic newborns and 148 symptomatic newborns (confirmed vertical sepsis: 31, vertical clinical sepsis: 38, non-infectious diseases: 79) were studied. In asymptomatic neonates, PCT values at 12–24 h were significantly higher than at birth and at 36–48 h of life. Resuscitation at birth and chorioamnionitis were independently associated to PCT values. Neonates with confirmed vertical sepsis showed significantly higher PCT values than those with clinical sepsis. PCT thresholds for the diagnosis of sepsis were 0.55 ng/mL at birth (sensitivity 75.4%, specificity 72.3%); 4.7 ng/mL within 12–24 h of life (sensitivity 73.8%, specificity 80.8%); and 1.7 ng/mL within 36–48 h of life (sensitivity 77.6%, specificity 79.2%).

**Conclusion:**

Serum PCT was moderately useful for the detection of sepsis of vertical transmission, and its reliability as a maker of bacterial infection requires specific cutoff values for each evaluation point over the first 48 h of life.

## Background

Neonatal sepsis of vertical transmission remains a major problem associated with high morbidity and mortality in newborns, particularly amongst preterm infants [1-4]. Rapid diagnosis is problematic because the first signs of this disease may be minimal, and are similar to those of various non-infectious processes; furthermore, bacterial cultures are time-consuming, and other laboratory tests are either not available for routine use or lack sensitivity or specificity. In this situation, neonates with risk factors for infection or clinical suspicion of infection are empirically treated with antibiotics. To avoid the unnecessary treatment of uninfected patients, an early, sensitive and specific laboratory test would be helpful to guide clinicians in neonatal units in deciding whether or not to start administering antibiotics. Several leukocyte indices and acute-phase protein levels have been evaluated for the diagnosis of sepsis, and more recently, measurement of multiple plasma cytokines [5] and leukocyte activation markers [6] have showed promising results. However, to date, no single laboratory test has provided rapid and reliable identification of early infected neonates. This inability has led to a search for new diagnostic markers [7,8].

It has been recently reported that procalcitonin (PCT), the prohormone of calcitonin, increases markedly in septic conditions [9] and it appears to be a good predictor of infection severity. Furthermore, the finding that PCT is released into the circulation within 3 h after endotoxin injection, plateaus at 6 h, and remains elevated for 24 h, makes PCT a promising new agent for early and sensitive identification of infected patients [10]. The results of recent studies suggest the usefulness of PCT for early diagnosis of neonatal sepsis [11-23], although other investigators have observed lack of accuracy for this marker [24-26]. Also, since markedly increased concentrations during the first 48 h of life in newborn infants without bacterial infection have been reported [12,23,27], there are conflicting data regarding what constitutes an abnormal value.

The objective of this study was to determine the behavior of serum PCT concentrations in uninfected and infected neonates, and to assess the value of this marker for the diagnosis of neonatal sepsis of vertical transmission.

## Methods

Since 1995 the neonatal services of 28 acute-care teaching hospitals distributed across 10 Autonomous Communities in Spain ("Grupo de Hospitales Castrillo") have been involved in an ongoing prospective surveillance project to assess the incidence and characteristics of infection of vertical transmission during the newborn period. The neonatal services of 13 hospitals participated in the present study. Between January 2000 and January 2001, all consecutive neonates within 48 h of life were prospectively included in the study if blood samples were available for timed PCT measurement according to three postnatal periods: shortly after birth, within 12–24 h of life, and within 36–48 h of age, and complete neonatal and outcome data were collected to classify the newborn infant into two distinct populations. Exclusion criteria were infants born to mothers with gestational diabetes (because available data indicate that PCT values are higher for neonates born to diabetic mothers [12]) and refusal of parental consent for blood sampling. The study was approved by the Ethics Committees of the participating hospitals and the parents gave their written informed consent.

Two distinct populations were defined. The first population (group 1) included a group of asymptomatic newborn infants admitted during the first 24 h of life to the neonatal unit because of prematurity, low birth weight, or at least two risk factors for infection (table 1). They had no clinical signs of sepsis during their first week of life and had a negative blood culture. Patients in this group did not receive antibiotic treatment. Those who were discharged home before the seventh day of live were followed to assure they did not develop late-onset vertical sepsis.

The second population included symptomatic neonates who were admitted to the neonatal unit and were evaluated for sepsis during the first 48 h of life. Patients in this population were later classified as group 2A (confirmed vertical sepsis), group 2B (vertical clinical sepsis), or group 2C (non-infectious disease). Criteria for confirmed vertical neonatal sepsis and vertical clinical sepsis were those previously established by "Grupo de Hospitales Castrillo" [1,2]. Clinical signs of neonatal sepsis and risk factors for vertical transmission are shown in Table 1.

- Group 2A: Confirmed vertical neonatal sepsis, defined as at least three clinical signs of infection in association with at least one bacteriological evidence of infection. Evidence of infection included positive blood culture, at least three positive surface swabs in the first 24 h of life for traditional pathogens of vertical transmission – such as group B streptococci (GBS) and *Escherichia coli*-, or GBS culture-positive mother).

- Group 2B: Vertical clinical sepsis, determined by at least three clinical signs of infection, at least one laboratory finding consistent with infection (leukocyte count >30,000 cells/μL or <5,000 cells/μL, C-reactive protein > 12 mg/L), negative blood culture, and at least two risk factors for vertical transmission and/or intrapartum administration of antibiotics.

- Group 2C: Uninfected newborn infants with neonatal pathology other than an infectious process and with negative blood culture.

### PCT assay

Blood samples were centrifuged within 30 min of collection. Serum was stored at -20°C before analysis. PCT was measured in duplicate by a specific immunoluminometric assay (LUMItest^®^, Brahms Diagnostica GmbH, Berlin, Germany), requiring 20 μL of serum and 2 h to complete. The detection limit of this immunoluminometric assay is 0.08 ng/mL. Luminescence was measured automatically on a Lumat LB 9507 tube luminometer (Berthold Technologies GmbH & Co. KG, Bad Wildbad, Germany). Intraassay coefficients of variation were 5.9% at 31.0 ng/mL and 13.8% at 0.81 ng/mL; interassay coefficients of variation were 10.8% and 25.9% for the same PCT concentrations.

All PCT assays were performed by two centralised clinical chemistry laboratories of two participating hospitals.

### Statistical analysis

Statistical analysis was done with SPSS version 11.0 (SPSS Inc., 2001, Chicago, Ill, USA) and EPIDAT version 3.0 (Servicio de Información sobre Saúde Pública de la Consellería de Sanidade e Servicios Sociais de la Xunta de Galicia, Spain, and the Health Situation Analysis Program of the Pan American Health Organization, Washington D.C., USA). PCT values are expressed as median and interquartile (25th–75th) ranges. Neither PCT values nor their log transformation were normally distributed according to kolmogorov-smirnov test, thus non-parametric tests were used for comparisons between groups (Kruskal-Wallis test and Mann-Whitney U test).

The association between different perinatal variables (cesarean section, rupture of membranes more than 18 h prior to delivery, clinical chorioamnionitis, resuscitation at birth, and prenatal steroid exposure) and PCT values in asymptomatic neonates was assessed with multiple linear regression analysis. As PCT variable was not normally distributed, it was log-transformed and then extreme values were removed, so the variable showed adequate homocedasticity for this analysis. In order to report the effect of the perinatal variables, the regression coefficients and 95% confidence limits were exponentiated. R-square values were calculated to estimate how well the model explained PCT variability.

The reliability of serum PCT concentration for the diagnosis of sepsis of vertical transmission was calculated by receiver-operating characteristics (ROC) curves. Youden's index (sensitivity + specificity - 1) was used for determination of optimal cutoff values of the diagnostic tests in the different postnatal periods. Sensitivity, specificity, and the likelihood ratio of positive and negative results with a 95% confidence interval (CI) were calculated. Statistical significance was set at *P *< 0.05.

## Results

The study included 827 blood samples collected from 317 neonates who met the inclusion criteria. Group 1 included 169 asymptomatic newborns. There were 148 symptomatic neonates distributed in group 2 as follows: 31 newborns with confirmed vertical sepsis (group 2A), 38 with vertical clinical sepsis (group 2B) and 79 in the group of non-infectious diseases (group 2C). Non-infectious diseases were all of respiratory origin (hyaline membrane disease: 60, transient respiratory distress of the newborn: 12, meconium aspiration syndrome: 5, pneumothorax: 2). Causative pathogens of confirmed vertical sepsis included GBS in 19 cases, *E. coli *in 8, *Streptococcus bovis *in 1, *Klebsiella oxytoca *in 1, *Haemophilus infuenzae *in 1, and *Candida albicans *in 1. Clinical and anthropometric characteristics are shown in Table 2. There were 2 deaths in group 2A, no deaths in group 2B, and 3 deaths in group 2C. Patients in group 2C had significantly lower birth weights and gestational ages (P < 0.0001, analysis of variance) than those in groups 1, 2A and 2B.

**Figure 1:**
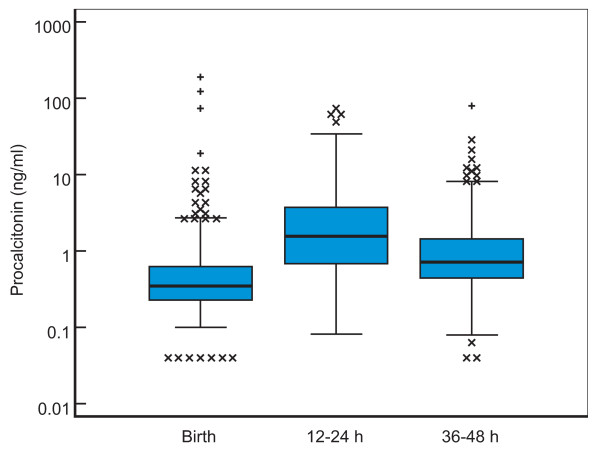
**Changes in PCT from birth to 48 h of life in asymptomatic newborns**. Box plot comparing serum PCT concentrations in asymptomatic newborns (group 1) at birth, at 12–24 h and 36–48 h of life. The boxes are limited by the 75^th ^and 25^th ^percentiles of the data. The middle lines represent the median. Values plotted with cross markers are outside values (more than 1.5 times the interquartile range over 75^th ^or under 25^th ^percentiles). Values plotter with plus markers are far out values (more than 3 times the interquartile range over 75^th ^or under 25^th ^percentiles).

Pairwise comparisons of serum PCT concentrations in group 1 at each postnatal period confirmed higher serum PCT values (median [interquartile range]) at 12–24 h of life (1.54 [0.68–3.78] ng/mL) than those at birth (0.35 [0.23–0.64] ng/mL, *P *< 0.0001, Mann-Whitney U test) and at 36–48 h of life (0.73 [0.45–1.48] ng/mL, *P *< 0.0001, Mann-Whitney U test) as shown in Figure 1.

Multiple linear regression analyses identified some variables independently associated to PCT values in group 1. PCT concentrations were associated with resuscitation at birth (defined as the necessity of tracheal intubation) at 36–48 h of life, and with chorioamnionitis at birth (Table 3). Nevertheless, variables specified in the model accounted for just a small part of the PCT variability (10.1% at birth, 2.0% at 12–24 h, and 9.4% at 36–48 h of life).

Serum PCT concentrations of patients in groups 2A, 2B, and 2C were higher than those of patients in group 1 at each postnatal period, as showed in Table 4 (*P *< 0.0001, Mann-Whitney U test). Patients of group 2A (confirmed vertical sepsis) had consistently higher serum PCT values than those of group 2B (vertical clinical sepsis) at birth (*P *= 0.006), at 12–24 h (*P *= 0.002), and 36–48 h of life (*P *= 0.032). There were no significative differences between the PCT concentrations of neonates with respiratory diseases (group 2C) and those with clinical sepsis.

**Figure 2:**
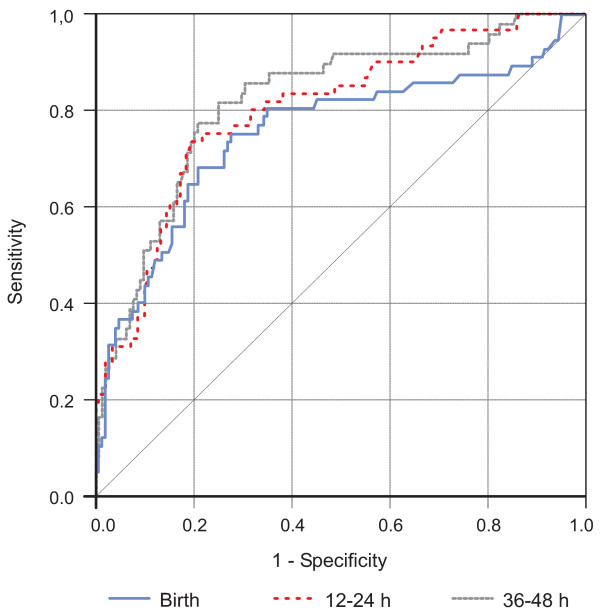
**ROC curves**. ROC curves of PCT at birth, and at 12–24 h and 36–48 h of life for the diagnosis of neonatal sepsis of vertical transmission.

We elaborated ROC curves considering groups 2A and 2B as infected and group 1 as non-infected. ROC of PCT at birth and 12–24 h and 36–48 h of life for the diagnosis of sepsis of vertical transmission are shown in Figure 2. The areas under the ROC curve for the three periods were 0.754 (95% CI, 0.671 to 0.838), 0.802 (95% CI, 0.735 to 0.869), and 0.820 (95% CI, 0.750 to 0.891), respectively. Cutoff levels with the optimum diagnostic efficiency derived from the ROC curves were ≥ 0.55 ng/mL at birth (sensitivity 75.4%, specificity 72.3%), ≥ 4.7 ng/mL at 12–24 h of life (sensitivity 73.8%, specificity 80.8%), and ≥ 1.7 ng/mL at 36–48 h of life (sensitivity 77.6%, specificity 79.2%) (Table 5). We also selected other cutoff points in order to get better sensitivity (>90%) as seen in Table 6.

## Discussion

This study shows that increased serum PCT concentrations in newborn infants are related to different factors other than neonatal sepsis. Elevated PCT levels were apparent 12–24 h after birth in asymptomatic uninfected infants. The physiological peak of serum PCT concentrations in healthy neonates has been previously reported [12,23], and increased PCT concentrations have also been found in neonates with very low probability of infection [27,28]. Although PCT may cross the placental barrier, the findings of higher PCT concentrations in cord sera compared with maternal samples, with even larger differences at 24 and 48 h of age, cannot be explained on the grounds of maternal transfer alone; therefore, the postnatal surge of PCT most likely represents endogenous synthesis [29]. This phenomenon might be attributed to direct stress on the baby during the perinatal period or to the adaptation to the extrauterine environment. The normal birth process and the extrauterine adaptation stimulates an acute phase reaction in the newborn infant with a release of C-reactive protein (CRP), interleukin-6 (IL-6), and serum amyloid A [30,31].

The higher concentrations of PCT observed in uninfected infants with respiratory disorders (mostly hyaline membrane disease) compared with asymptomatic infants is also consistent with previous reports. Other authors have shown elevated PCT values during the first 10 days of life in uninfected newborns who presented respiratory distress syndrome (RDS) [26,32]. Because no significant effect related to the severity of RDS could be detected, Monneret et al. [32] suggested that hypoxemia (a common event to each etiology of RDS and which is transient during delivery) could be responsible for these increased PCT values, providing further support for the hypothesis of pulmonary PCT synthesis. Yet it is not possible to assure these patients were really uninfected, since evidence of no infection was based on negative bacterial cultures, absence of fever, and a normal CRP value, and these criteria may fail in an appreciable number of cases [33]. Our study has a similar problem, as intrapartum antibiotics had been used in 22/79 neonates with respiratory disorders, and 66/79 were treated with antibiotics after birth. This is also the reason why we excluded group 2C from ROC curves.

Elevated PCT levels in newborn infants who required resuscitation at birth may be related to the stressful situation or tissular hypoxia. Similar findings have been reported in perinatal asphyxia [32] and need for delivery room intubation [13]. Chorioamnionitis has been also independently associated with increased PCT values [13]. These findings in our study must be considered cautiously, since variables selected for the multiple linear regression analysis explained just a small piece of total PCT variability. It is noteworthy that we found no effect of perinatal variables on PCT at 12–24 h of life, when PCT values were higher.

In this study, serum PCT concentrations showed a moderate diagnostic efficiency in the detection of sepsis of vertical transmission. When considering a potentially serious illness as neonatal sepsis, the selection of cutoff values for a diagnostic marker must emphasize sensitivity over specificity [34]. But with our ROC curves, improvements in sensitivity implied big reductions in specificity, and the overall efficacy was quite better using Youden's index to select the cutoff points (Tables 5 and 6). Specific cutoff values for each evaluation point over the first 48 h of life were needed to improve the diagnostic accuracy of this marker of bacterial infection. In 1998, Chiesa et al. [12] determined reference ranges for PCT across the range of postnatal hours from 0 to 48 h and, in a further study, these authors showed that PCT sensitivity and specificity were greater than those of CRP or interleukin 6 if different cutoff points at birth and at 24 h and 48 h of life were used [13]. The sensitivity and specificity values found in our study according to specific cutoff points for each evaluation point over the first 48 h of life are within the range of 57–100% sensitivity and 50–100% specificity previously reported for thresholds ranging between 0.5 and 100 ng/mL [9,11-17,24-26]. The heterogeneity in the study design, particularly the definition of sepsis, and the different nature of the control groups might explain the differences in sensitivity and specificity. Neonatal sepsis of vertical transmission was diagnosed according to criteria defined by our group for infections diagnosed within the first 3 days after delivery [1,2]. However, the present results refer to early-onset neonatal sepsis because the study population was limited to neonates within 48 h of life.

A limitation of this study is that the reliability of PCT was not compared with CRP or other infection markers, such the leukocyte count, because these analyses were not standardised and performed at every biochemical laboratory of the participating hospitals. Some studies have shown that PCT is more reliable than CRP as a test for the diagnosis of early-onset neonatal sepsis [13-15], but other studies have not found any advantage of PCT over CRP [11,23-25]. However, it is important to consider that large sample sizes are needed to assess differences in the accuracy of diagnostic tests, e.g., according to an estimated 25% prevalence of sepsis and an absolute difference of 15% between sensitivities of PCT and CRP, approximately 300 patients would be needed for 80% statistical power. Another limitation of our study is that we used a control group formed by asymptomatic infants without evidence of infection, which may overestimate the reliability of PCT as diagnostic test when used in a population of neonates with clinical suspicion of sepsis [35].

To be able to establish precise conclusions regarding the usefulness of PCT and other inflammatory response markers, some methodological issues should be solved [36], specially regarding a sufficiently reliable "gold standard" of neonatal sepsis. Although a recent international consensus conference has adapted diagnostic criteria for sepsis to several pediatric age groups, including newborn infants, premature infants were not included [37]; so these criteria for sepsis must be evaluated before being used in the neonatal intensive care unit. On the other side, although, in the field of neonatology, the concept of "clinical sepsis" is widely used, a uniform definition of this common diagnosis is lacking, as is a standard criterion for the inclusion or exclusion of probable/suspected sepsis from the analysis [38]. Moreover, the risk of neonatal sepsis is likely to be different with varying scenarios related to the neonatal population [7]. An optimal design of these studies should allow assessing the pre-test probability of sepsis according to different clinical features and risk factors for infection.

## Conclusion

We conclude that the serum PCT concentration showed a moderate diagnostic value for the detection of sepsis of vertical transmission, with better results after 12 h of birth. The reliability of PCT as a maker of bacterial infection requires specific cutoff values for each evaluation time point beyond the first 48 h of life.

## Abbreviations

CI: confidence interval.

CRP: C-reactive protein.

EPIDAT: epidemiological analysis of tabulated data.

GBS: group B streptococci

GHC: Grupo de Hospitales Castrillo

PCT: procalcitonin.

RDS: respiratory distress syndrome

ROC: receiver-operating characteristics.

## Competing interests

The author(s) declare that they have no competing interests.

## Authors' contributions

JBLS had primary responsibility for protocol development, outcome assessment, preliminary data analysis and writing of the manuscript.

DPS participated in the development of the protocol, literature search, performed the final data analyses, and had primary responsibility for writing of the manuscript.

VRS had primary responsibility for protocol development, patient enrolment and preliminary data analysis, supervised the design and execution of the study, and revised the manuscript for scientific content.

BFC and GDCC participated in the development of the protocol, patient enrollment, and contributed to the writing of the manuscript.

Members of the GHC who participated in the development of the protocol and patient enrollment are listed in the Acknowledgements section.

## Pre-publication history

The pre-publication history for this paper can be accessed here:



## Figures and Tables

**Table 1:** Clinical signs of neonatal sepsis and risk factors for vertical transmission

Clinical signs	Data
Respiratory	Tachypnea (> 70 breaths/min in preterm babies, > 60 breaths/min in neonates at term)
	Subcostal and/or intercostal retractions
	Grunting
	Apnea > 10 seconds
Haemodynamic	Cyanosis
	Pallor
	Hypotension (blood pressure < 2 SD of the mean for age and weight)
	Tachycardia (> 180 beats/min)
	Bradichardia (< 100 beats/min in preterm babies, < 80 beats/min in neonates at term)
Digestive	Rejection of food
	Vomiting
	Abdominal distention
	Hepatomegaly
	Poor peripheral perfusion
Haematological	Anaemia (haemoglobin < 10 g/dL in preterm babies, < 11 g/dL in neonates at term)
	Jaundice
	Petechiae
	Echymoses
Neurological	Hypotony
	Irritability
	Letargy
	Convulsions
Risk factors for infection	Spontaneous premature delivery
	Time of membrane rupture > 18 h
	Intrapartum maternal fever > 38°C
	Clinical chorioamnionitis (two of the following criteria: maternal fever, foetal tachycardia >140 bpm, purulent or foul-smelling amniotic fluid, uterine tenderness)
	Previous offspring with GBS infection
	Maternal group B streptococci colonisation
	Prenatal use of corticosteroids

**Table 2:** Obstetric history and somatometric characteristics of patients included in the study

	Group 1	Group 2A	Group 2B	Group 2C
No. patients	169	31	38	79
Gestational age, weeks, mean (SD)	36.2 (3.7)	34.5 (5.4)	35.2 (4.8)	30.3 (6.2)
Weight at birth, g., mean (SD)	2622 (860)	2496 (1107)	2573 (997)	1542 (880)
GBS colonisation, no. (%)	44 (26.0)	13 (41.9)	0 (0)	11 (13.9)
Prenatal steroid exposure, no. (%)	40 (23.7)	9 (29.0)	9 (23.7)	38 (48.1)
Membrane rupture > 18 h, no. (%)	70 (41.4)	18 (22.8)	5 (13.2)	11 (35.5)
Intrapartum fever, no. (%)	15 (8.9)	7 (22.6)	8 (21.1)	3 (3.8)
Chorioamnionitis, no. (%)	10 (5.9)	4 (12.9)	3 (7.9)	8 (10.1)
Intrapartum antibiotics, no. (%)	105 (62.1)	15 (48.4)	12 (31.6)	22 (27.8)
Resuscitation at birth, no. (%)	10 (5.9)	8 (25.8)	8 (21.1)	33 (41.8)
Males, no. (%)	93 (55.0)	15 (48.4)	16 (42.1)	43 (54.4)

Group 1: asymptomatic newborn infants; group 2A: confirmed vertical neonatal sepsis; group 2B: vertical clinical sepsis; group 2C: neonatal pathology other than an infectious process (respiratory diseases).

**Table 3:** Independent effects of some perinatal variables on serum procalcitonin values at birth, at 12–24 h and 36–48 h of life.

Risk factor	Serum PCT values, ng/mL, median (interquartile 25th–75th range)		
	
	At birth	12–24 h of life	36–48 h of life
Cesarean section	1.08 (0.58–2.02)	0.73 (0.26–2.02)	0.91 (0.54–1.51)
Membrane rupture > 18 h	0.67 (0.37–1.20)	1.32 (0.51–3.45)	0.96 (0.59–1.56)
Chorioamnionitis	18.61 (2.43–142.5)*	1.08 (0.14–8.15)	1.78 (0.54–5.84)
Resuscitation at birth	1.30 (0.39–4.40)	5.58 (0.49–63.17)	8.71 (2.40–31.57)*
Prenatal steroid exposure	1.84 (0.91–3.73)	0.58 (0.17–1.95)	0.93 (0.49–1.76)

Includes data of 169 asymptomatic newborns (group 1). Data are expressed as regression coefficients (95% confidence interval).

* *P *< 0.01.

**Table 4:** Serum procalcitonin values at birth, at 12–24 h and 36–48 h of life in the groups included in the study.

Study group	Serum PCT values, ng/mL median (interquartile 25th–75th range)		
	
	At birth	12–24 h of life	36–48 h of life
Group 1, n = 169	0.35 (0.23–0.64)	1.54 (0.68–3.78)	0.73 (0.45–1.48)
Group 2A, n = 31	13.59 (0.50–101.3)	30.65 (6.38–95.9)	6.74 (2.85–29.15)
Group 2B, n = 38	1.09 (0.54–3.04)	5.88 (1.53–11.92)	2.52 (1.44–8.71)
Group 2C, n = 79	1.12 (0.38–7.06)	4.79 (1.22–16.89)	2.96 (0.97–7.11)

Group 1: asymptomatic newborn; group 2A: confirmed vertical neonatal sepsis; group 2B: vertical clinical sepsis; group 2C: neonatal pathology other than an infectious process (respiratory diseases).

**Table 5:** Performance of procalcitonin at birth, at 12–24 h, and 36–48 h of life for the diagnosis of neonatal sepsis of vertical transmission (cutoff points selected according to Youden's index).

	At birth n = 205	12–24 h of life n = 212	36–48 h of life n = 193
Cutoff, ng/ml	≥0.55	≥4.7	≥1.7
Sensitivity, % (95% CI)	75.4 (62.9 – 84.8)	73.8 (61.6 – 83.2)	77.6 (64.1 – 87.0%)
Specificity (95% CI)	72.3 (64.6 – 78.9)	80.8 (73.8 – 86.3)	79.2 (71.8 – 85.0)
Youden's index	0.48	0.55	0.57
PPV (95% CI)	51.2 (40.7 – 61.6)	60.8 (49.4 – 71.1)	55.9 (44.1 – 67.1)
NPV (95% CI)	88.4 (81.5 – 93.0)	88.4 (82.0 – 92.7)	91.2 (84.9 – 95.0)
LR+ (95% CI)	2.72 (2.02 – 3.67)	3.84 (2.68 – 5.50)	3.72 (2.62 – 5.29)
LR- (95% CI)	0.34 (0.21 – 0.55)	0.32 (0.21–0.50)	0.28 (0.17 – 0.48)

PPV: positive predictive value, NPV: negative predictive value, LR+: likelihood ratio of a positive result, LR-: likelihood ratio of a negative result.

**Table 6:** Performance of procalcitonin at birth, at 12–24 h, and 36–48 h of life for the diagnosis of neonatal sepsis of vertical transmission (cutoff points selected to achieve 90% sensitivity).

	At birth n = 205	12–24 h of life n = 212	36–48 h of life n = 193
Cutoff, ng/ml	≥0.15	≥1.2	≥0.75
Sensitivity, % (95% CI)	91.2 (81.1 – 96.2)	90.2 (80.2 – 95.4)	91.8 (80.8 – 96.8%)
Specificity (95% CI)	10.8 (6.8 – 16.8)	43.0 (35.4 – 51.0)	51.4 (43.3 – 59.4)
Youden's index	0.02	0.33	0.43
PPV (95% CI)	28.3 (22.3 – 35.2)	39.0 (31.3 – 47.2)	55.9 (44.1 – 67.1)
NPV (95% CI)	76.2 (54.9 – 89.4)	91.5 (82.8 – 96.1)	91.2 (84.9 – 95.0)
LR+ (95% CI)	1.02 (0.93 – 1.13)	1.58 (1.35 – 1.86)	1.89 (1.57 – 2.28)
LR- (95% CI)	0.81 (0.32 – 2.07)	0.23 (0.10–0.50)	0.16 (0.06 – 0.41)

PPV: positive predictive value, NPV: negative predictive value, LR+: likelihood ratio of a positive result, LR-: likelihood ratio of a negative result.
